# Fiske Fund Prize Questions

**Published:** 1844-11-16

**Authors:** 


FISKE FUND PRIZE QUESTIONS.

The Trustees of the Fiske Fund, in Rhode Island, propose the following questions for 1844–45:—1. "The best mode of treating, and the best apparatus for the management of, fractures of the thigh." 2. "The character, causes and best treatment of bronchitis." For the best dissertation on each of these questions, the sum of fifty dollars will be paid—the dissertations to be sent, previous to May 10, 1845, to Dr. L. L. Miller, of Providence, Dr. T. C. Dunn, of Newport, or Dr. Jabez Holmes, of Bristol.—*N. Y. Journal of Medicine.*

